# New players tip the scales in the balance between excitatory and inhibitory synapses

**DOI:** 10.1186/1744-8069-1-12

**Published:** 2005-03-23

**Authors:** Joshua N Levinson, Alaa El-Husseini

**Affiliations:** 1 Department of Psychiatry, The Brain Research Centre, University of British Columbia, Vancouver, British Columbia, V6T 1Z3, Canada

## Abstract

Synaptogenesis is a highly controlled process, involving a vast array of players which include cell adhesion molecules, scaffolding and signaling proteins, neurotransmitter receptors and proteins associated with the synaptic vesicle machinery. These molecules cooperate in an intricate manner on both the pre- and postsynaptic sides to orchestrate the precise assembly of neuronal contacts. This is an amazing feat considering that a single neuron receives tens of thousands of synaptic inputs but virtually no mismatch between pre- and postsynaptic components occur *in vivo*. One crucial aspect of synapse formation is whether a nascent synapse will develop into an excitatory or inhibitory contact. The tight control of a balance between the types of synapses formed regulates the overall neuronal excitability, and is thus critical for normal brain function and plasticity. However, little is known about how this balance is achieved. This review discusses recent findings which provide clues to how neurons may control excitatory and inhibitory synapse formation, with focus on the involvement of the neuroligin family and PSD-95 in this process.

## 

In the brain, excitatory and inhibitory synaptic transmission is mainly mediated by two neurotransmitters: glutamate which is released at excitatory glutamatergic synaptic contacts, and γ-amino butyric acid (GABA) which is released at inhibitory GABAergic synapses. Neural information processing is believed to be mediated by integration of excitatory and inhibitory synaptic inputs [1-3]. Therefore, precise controls must exist to maintain an appropriate number of one type of synaptic input relative to the other. This process is thought to be governed by homeostatic feedback mechanisms, however factors involved remain elusive [4,5]. Impressive work carried out in recent years has begun to address the roles of molecules involved in synapse formation. A theme that has emerged from these studies is that glutamatergic and GABAergic synapses consist of complex, yet distinct networks of proteins on the postsynaptic side. The major challenge in this field now is to understand how this molecular machinery is involved in synapse formation and specificity.

## What controls excitatory synapse development?

The discovery of a protein complex that regulates postsynaptic glutamate receptor clustering and the formation of dendritic spines has revealed some of the mechanisms involved in excitatory synapse development. Two main groups of key regulators of excitatory synapse formation have been identified, namely postsynaptic scaffolding proteins and cell adhesion molecules (CAMs). In the first group, several proteins including members of the PSD-95 family, shank, and homer have been shown to promote excitatory synapse maturation (reviewed in [6]). Much work has focused on postsynaptic density protein-95 (PSD-95), one of the most abundant proteins in the PSD [6]. PSD-95 clustering at synapses occurs early in development, prior to other postsynaptic proteins [7], and *discs large*, a *Drosophila *homolog of PSD-95, is required for normal neuromuscular junction development in larva [8]. In addition, PSD-95 enhances AMPA-type glutamate receptor clustering and activity through interaction with stargazin [9,10]. The second group, CAMs, have long been implicated in the formation of cell-cell contact, however the roles of CAMs in the initiation and stabilization of excitatory synaptic contacts have only recently been discovered [11]. CAMs interact transsynaptically through homophilic interactions, such as in the case of SynCAM 1 and protocadherins, or through heterophilic binding, such as with neuroligin and its binding partner, β-neurexin. It remains unresolved whether different sets of CAMs cooperate to modulate synaptic stability and specificity.

## New players in inhibitory synapse formation

Although much progress has been made with respect to factors involved in the formation of excitatory synapses, molecules that control inhibitory synapse formation have remained largely unknown. Gephyrin, a scaffolding protein enriched at inhibitory synapses, is one of a small number of proteins that modulate GABA receptor clustering [12]. Also, the neural CAMs L1, dystroglycan and L-CAM have been indirectly implicated in the establishment of inhibitory synapse formation, however further work is needed to clarify their involvement in this process [13-15].

**Figure 1:**
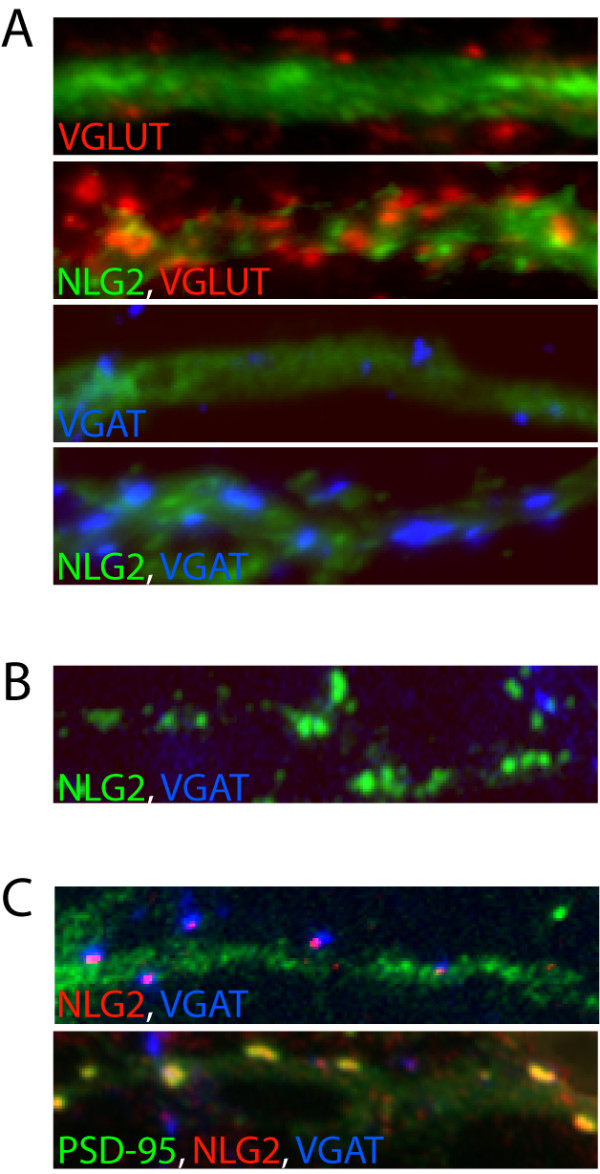
Neuroligins, β-neurexin, and PSD-95 modulate excitatory and inhibitory synapse formation. An example of the effects of a member of the neuroligin (NLG) family, NLG2 (green), on synapse formation. (A) Expression of NLG2 in hippocampal neurons increases the number of excitatory (VGLUT-positive; red) and inhibitory (VGAT-positive; blue) presynaptic contacts. (B) Interfering with β-neurexin and NLG2 coupling blocks NLG2 (green)-mediated effects on inhibitory synapse formation. Treatment with a soluble form of β-neurexin decreases the number of sites positive for VGAT (blue). (C) NLG2 (red) is normally localized at inhibitory synaptic contacts (VGAT-positive; blue; upper panel). Overexpression of PSD-95 shifts NLG2 from inhibitory to excitatory (PSD-95-positive; green) synapses (colocalization of NLG2 and PSD-95 appears in orange; lower panel).

New findings from Prange et al. (2004) shed some light on the involvement of members of the neuroligin (NLG) family of adhesion molecules in inhibitory synapse formation [16]. Unexpectedly, overexpression of NLG1 induced not only excitatory synapses but also robustly increased the number and size of inhibitory presynaptic terminals. The effect on inhibitory synapses was not restricted to NLG1, as NLG2 and NLG3 were capable of inducing similar effects on both excitatory and inhibitory presynaptic terminals (an example of the effects of NLG2 can be seen in Fig. 1A) [17]. Similar results were recently reported by Chih et al. (2005) [18]. If this is physiologically relevant, one would expect members of the NLG family to be localized at both excitatory and inhibitory synapses. Indeed, work done by Brose and co-workers was the first to resolve part of this mystery, reporting that NLG2 is concentrated at inhibitory synapses [19]. Later studies reported similar observations on the enrichment of NLG2 at inhibitory synapses [17,18,20]. This is in contrast to NLG1, which is enriched at excitatory synapses [21].

## How do neuroligins mediate excitatory and inhibitory synapse formation?

NLG1 was originally identified as a binding partner of the presynaptic cell adhesion molecule, β-neurexin, which is known to be coupled to a presynaptic protein complex [22-24]. Thus, coupling of NLGs to β-neurexin may activate an array of molecular responses leading to the structural reorganization of the presynaptic compartment. In support of this, a soluble form of β-neurexin blocks the formation of presynaptic terminals induced by heterologously expressed NLG1 [25]. Another important finding by Graf et al. (2004) showed that β-neurexin expressed in non-neuronal cells or coupled to beads is sufficient to induce the differentiation of inhibitory postsynaptic sites [20]. These results are further supported by experiments in hippocampal neurons which showed that inhibitory synapses induced by NLG1 and NLG2 can be blocked by soluble β-neurexin [17]. An example of the effects of soluble β-neurexin on NLG2-mediated inhibitory synapse formation is shown in Fig. 1B. Together, this provides a novel mechanism for inhibitory synapse formation mediated through NLG-β-neurexin coupling. However, it remains unclear how the interaction between NLGs and β-neurexin regulate synapse specificity since, β-neurexin can mediate the formation of both excitatory and inhibitory synapses.

## Controlling the balance between excitatory and inhibitory synapses

A critical finding depicted from recent work by Prange et al. (2004) shows that association of NLGs with scaffolding proteins may control the balance between excitatory and inhibitory synapses [16]. PSD-95 is known to bind NLG1 and recruit it to synapses via its PSD-95/Dlg/ZO-1 homology (PDZ) domain [22,26,27]. As described above, expression of NLG1 alone induces the formation of both excitatory and inhibitory synapses. However, when coexpressed with PSD-95, NLG1 effects were restricted to excitatory synapses. Another intriguing finding is that overexpression of PSD-95 redistributes endogenous NLG2 from inhibitory to excitatory synapses (Fig. 1C) [17]. Presumably this occurs through association with the C-terminal PDZ-binding motif in NLG2. This correlates with the observation that PSD-95 overexpression enhances formation of excitatory synapses with a corresponding decrease in inhibitory synapse formation [16]. Such effects resulted in an overall increase in the excitatory to inhibitory (E/I) synapse ratio. A recent study by Chih et al. (2005) further supports the notion that NLGs are involved in regulating the E/I ratio [18]. Knockdown of NLGs, either individually or collectively, results in a substantial decrease in inhibitory synaptic transmission, with relatively little effect on transmission at excitatory synapses, thus altering the E/I synaptic balance.

**Figure 2:**
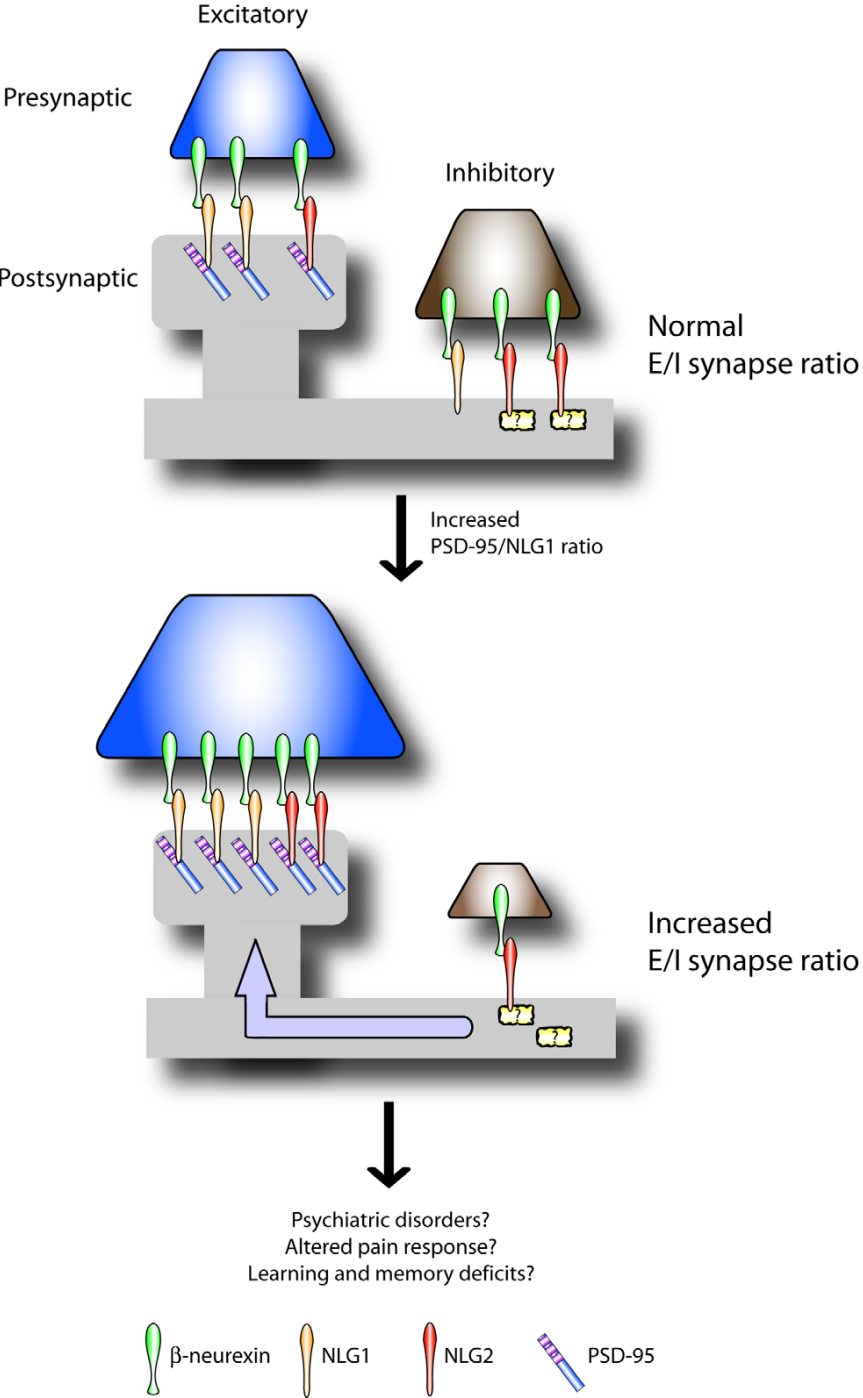
Relative levels of scaffolding proteins and cell adhesion molecules control the balance between excitatory and inhibitory synapses. NLGs and PSD-95 are used here as an example to demonstrate this concept. Under normal conditions, NLG1 is enriched at excitatory contacts whereas NLG2 is concentrated at inhibitory synapses. PSD-95 retains the majority of NLG1 at excitatory synaptic sites, whereas NLG2 localization is primarily controlled through interaction with an unknown scaffolding protein specific to inhibitory synapses. An increase in the levels of PSD-95 results in a shift of NLG2 molecules from inhibitory to excitatory synapses, presumably through PDZ-mediated binding to PSD-95. The resulting effect is an overall increase in the number of excitatory relative to inhibitory synapses, and thus an enhanced excitatory to inhibitory (E/I) synaptic ratio (for simplicity, changes in synapse number are indicated by changes in the size of the illustrated presynaptic terminals). An altered E/I ratio may result in defects in brain circuitry associated with behavioral and cognitive abnormalities such as those linked to psychiatric, pain response, and learning and memory disorders.

The changes observed upon manipulation of the levels of PSD-95 and NLGs provide new clues to the mechanisms involved in controlling the E/I ratio. Thus, a new model emerges; factors that regulate expression and stoichiometry between cell adhesion molecules and scaffolding proteins may be central to the formation of excitatory and inhibitory synapses and the control of E/I ratio (Fig. 2). In this model, all members of the NLG family can induce both excitatory and inhibitory synapses. However, PSD-95, and possibly other postsynaptic scaffolding proteins regulate targeting and/or retention of specific NLGs to a particular synaptic site, controlling which synapse type is induced by which NLG family member. This may therefore create a situation in which scaffolding proteins cooperate or compete with one another for directing individual members of the NLG family to a specific synapse type.

## Implications in neurodevelopmental abnormalities

Several physiological and pathological paradigms alter the levels of PSD-95. For example, PSD-95 association with the PSD is dynamic and is regulated by synaptic activity and palmitate cycling on PSD-95 [28]. Synaptic activity also upregulates PSD-95 expression through a neuregulin mediated pathway [29]. In contrast, administration of cocaine, a drug known to cause hyperexcitability, results in down regulation of PSD-95 in the striatum, a region mainly composed of inhibitory neurons [30]. Moreover, mutation of FMRP, a gene associated with fragile X mental retardation, results in a loss of regulation of PSD-95 expression [31]. The following question arises: Are alterations in the levels of certain postsynaptic scaffolding proteins or cell adhesion molecules sufficient to manipulate the E/I synapse ratio? One would expect that paradigms that interfere with proper assembly or expression of proteins that control E/I ratio may have drastic effects on synaptic balance if these changes occur during a period of active synapse formation.

A change in the E/I synapse balance has been proposed to be affected in many neurodevelopmental psychiatric disorders, including autism and some forms of mental retardation [32]. In particular, it is thought that autism is associated with enhanced E/I neurotransmission due to either increased excitation or reduced inhibition, and that this enhanced excitability leads to disruption of memory formation and abnormal social behaviour associated with this disorder. A potential defect in E/I ratio in autism and related disorders is emphasized by the recent discovery that frame shift mutations in the NLG3 and NLG4 genes, which result in early protein truncation and misfolding, are associated with autism [33-36]. In addition, chromosomal rearrangements in regions that harbor the NLG1, NLG2 and PSD-95 genes have also been implicated in autism [37-39]. The potential involvement of NLG genes as well as PSD-95 in autism therefore provides a possible molecular basis for this imbalance in E/I ratio, which manifests itself as abnormalities in patients affected with neurodevelopmental psychiatric disorders. Despite these exciting observations however, recent genetic screens suggest that mutations in NLGs are fairly rare in autism [40,41]. Therefore, it is more likely that neurodevelopmental psychiatric disorders may result from abnormal expression of a diverse set of genes with functions related to those of NLGs and PSD-95. In the adult brain, formation of new synaptic contacts is far less common, and thus CAMs and scaffolding proteins may be involved in controlling synaptic activity rather than synapse number. Alterations in the amounts of these proteins may therefore result in weakening or strengthening of either excitatory or inhibitory synaptic activity and in turn modulate the E/I balance.

## Conclusion

New findings provide evidence for a potential mechanism that controls the development of excitatory and inhibitory synapses, which at least partially involves synaptic cell adhesion and scaffolding molecules, among which are the NLG family of proteins and PSD-95. The levels of certain postsynaptic molecules relative to others appears to control the balance between different synapse types, and thus generation of a specific E/I ratio. This has important implications in neurodevelopmental disorders. To further understand how the E/I synaptic balance is established and maintained, it will be essential to address other issues. For instance, is synaptic activity involved in this process? If so, processes ranging from learning and memory to nociceptive transmission in the spinal cord, both of which are linked to neuronal activity, may be tied to control of E/I balance. To what extent does cross-talk between the pre- and postsynaptic sides play a role? At what developmental stage is this balance first established, and when does its stabilization occur? Despite these questions which remain unanswered so far, a staggering amount of progress has been made in this field in recent years. Surely, the excitement generated from this progress will lead to a more complete understanding of control of synaptic balance in the years to come.

## List of abbreviations

PSD-95, postsynaptic density protein-95; PDZ, PSD-95/Dlg/ZO-1 homology; NLG, neuroligin; GABA, γ-amino butyric acid; CAM, cell adhesion molecule; VGLUT, vesicular glutamate transporter; VGAT, vesicular GABA transporter.
